# A longitudinal study on morpho-genetic diversity of pathogenic *Rhizoctonia solani* from sugar beet and dry beans of western Nebraska

**DOI:** 10.1186/s12866-020-02026-9

**Published:** 2020-11-17

**Authors:** Saurav Das, T. Plyler-Harveson, Dipak K. Santra, Bijesh Maharjan, Kathy A. Nielson, Robert M. Harveson

**Affiliations:** Panhandle Research and Extension Centre, University of Nebraska-Lincoln, Scottsbluff, NE USA

**Keywords:** *Rhizoctonia solani*, Sugar beet, Dry bean, DNA marker, Nebraska, USA

## Abstract

**Background:**

Root and stem rot caused by *Rhizoctonia solani* is a serious fungal disease of sugar beet and dry bean production in Nebraska. *Rhizoctonia* root rot and crown rot in sugar beet and dry bean have reduced the yield significantly and has also created problems in storage. The objective of this study was to analyze morpho-genetic diversity of 38 *Rhizoctonia solani* isolates from sugar beet and dry bean fields in western Nebraska collected over 10 years. Morphological features and ISSR-based DNA markers were used to study the morphogenetic diversity.

**Results:**

Fungal colonies were morphologically diverse in shapes, aerial hyphae formation, colony, and sclerotia color. Marker analysis using 19 polymorphic ISSR markers showed polymorphic bands ranged from 15 to 28 with molecular weight of 100 bp to 3 kb. Polymorphic loci ranged from 43.26–92.88%. *Nei* genetic distance within the population ranged from 0.03–0.09 and Shannon diversity index varied from 0.24–0.28. AMOVA analysis based on ΦPT values showed 87% variation within and 13% among the population with statistical significance (*p* < 0.05). Majority of the isolates from sugar beet showed nearby association within the population. A significant number of isolates showed similarity with isolates of both the crops suggesting their broad pathogenicity. Isolates were grouped into three different clusters in UPGMA based cluster analysis using marker information. Interestingly, there was no geographical correlation among the isolates. Principal component analysis showed randomized distribution of isolates from the same geographical origin. Identities of the isolates were confirmed by both ITS-rDNA sequences and pathogenicity tests.

**Conclusion:**

Identification and categorization of the pathogen will be helpful in designing integrated disease management guidelines for sugar beet and dry beans of mid western America.

**Supplementary Information:**

The online version contains supplementary material available at 10.1186/s12866-020-02026-9.

## Background

**Fig. 1:**
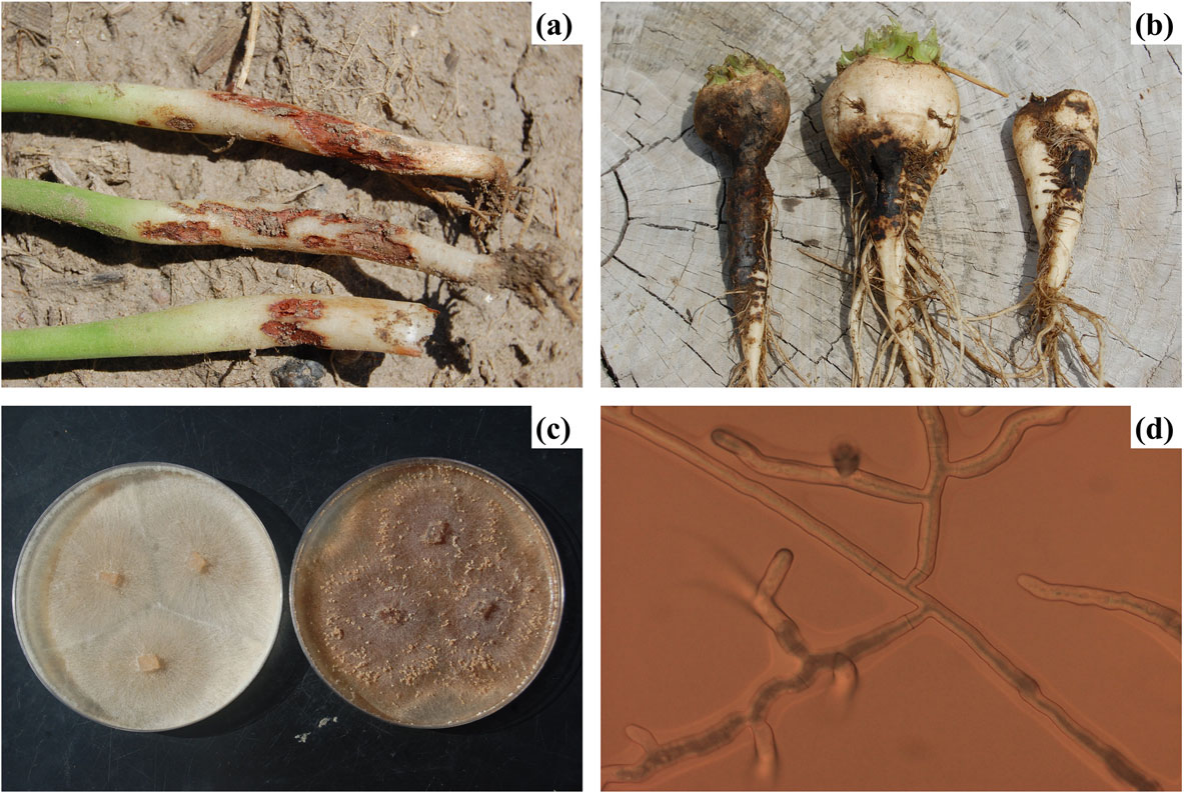
**a** Dry bean root rot (**b**) Sugar beet root rot (**c**) PDA culture plate (**d**) microscopic hyphal structure

*Rhizoctonia solani* is a polyphagous plant pathogen with worldwide distribution. It is a soil-borne pathogen and known for severe plant diseases like collar rot, root rot, damping off and wire stem [1]. The fungus survives on the infected plant debris and acts as an inoculum for the susceptible crops like sugar beet (*Beta vulgaris subsp. vulgaris*) [2], dry beans (*Phaseolus vulgaris*) [2], potato (*Solanum tuberosum*) [3], and soybean (*Glycine max)* [4]. It is a major problem for the sugar beet and dry bean producers of western Nebraska. Total production acreage of dry bean and sugar beet crops in Nebraska are 45,500 and 140,000–200,000 acres respectively. However, every year *Rhizoctonia* root rot and crown rot in sugar beet (Fig. 1b) and dry bean (Fig. 1a) have reduced the yield significantly and also created problems in storage. It has been estimated that on average 20% of annual sugar beet yield loss is due to the *Rhizoctonia* root and crown rot, and even in some rare scenarios 30–60% to complete loss of the crop has also been observed [5]. In Nebraska, a total of 52 and 42% of yield reduction can be observed in case of dry bean variety viz. Great Northern bean and Pinto bean, respectively due to Rhizoctonia root and crown rot [6].

*R. solani* occurs in varying levels of morphogenetic diversity. Cultural appearance, anastomosis, virulence, and physiology are different among different strains. Many scientific attempts have been made to categorize the *R. solani* isolates based on morphological, physiological and pathological differences. The most accepted grouping of *R. solani* is based on the formation of anastomosis or hyphal fusion [7, 8]. There are now 14 anastomosis groups (AG), several of which are divided into subgroups [9]. But the presence of more than one AG and occasional loss of anastomosis ability always complicated the identification and characterization of *Rhizoctonia sp.* Further, several studies have also indicated distinct pathogenesis even within the same AG groups [7, 10]. Morphological characteristics are further influenced by the culture conditions, which makes it more difficult to characterize and categorize the isolates. The problem associated with characterization can be better addressed with the DNA-based molecular studies [10–13]. Several DNA-based markers were used to analyze the genetic diversity of *R. solani.* These are genome sequence complementary analysis [14], random amplified polymorphic DNA (RAPD) [10, 13, 15], amplified fragment length polymorphism (AFLP) [16], simple sequence repeats (SSR) [17], and inter-simple sequence repeats (ISSR) [13, 18–21].

ISSR markers were developed in 1994 and since then these were widely used for rapid differentiation among the closely related species. The technique involves the amplification of the inter-region between two SSR regions with a primer of 16–18 bp long and with a flanking region of nucleotides at the 3′ or 5′ end. ISSR analysis is simple and less expensive than RAPD and AFLP. It can be used to assess the genetic diversity of a large number of phytopathogens within relatively less time and with high reproducibility [15]. Several researchers used dominant nature of the ISSR to establish the genetic variations and relationships among the *R. solani* isolates of different geographic regions and within the same anastomosis group [10, 13, 15].

Rhizoctonia root, stem, and crown rot are common in sugar beet and dry bean fields every year across western Nebraska. Our hypothesis was that isolates collected from two different crops could be different and also isolates collected from the same crop across the years and geographic region could be different. The objective of this study was to determine the morpho-genetic diversity among *R. solani* isolates of sugar beet (*RZ*_SB) and dry bean (*RZ*-DB) from western Nebraska. The genetic diversity was assessed with 19 polymorphic ISSR markers using a collection of isolates from the central great plains.

## Results

### Morphological diversity

**Fig. 2:**
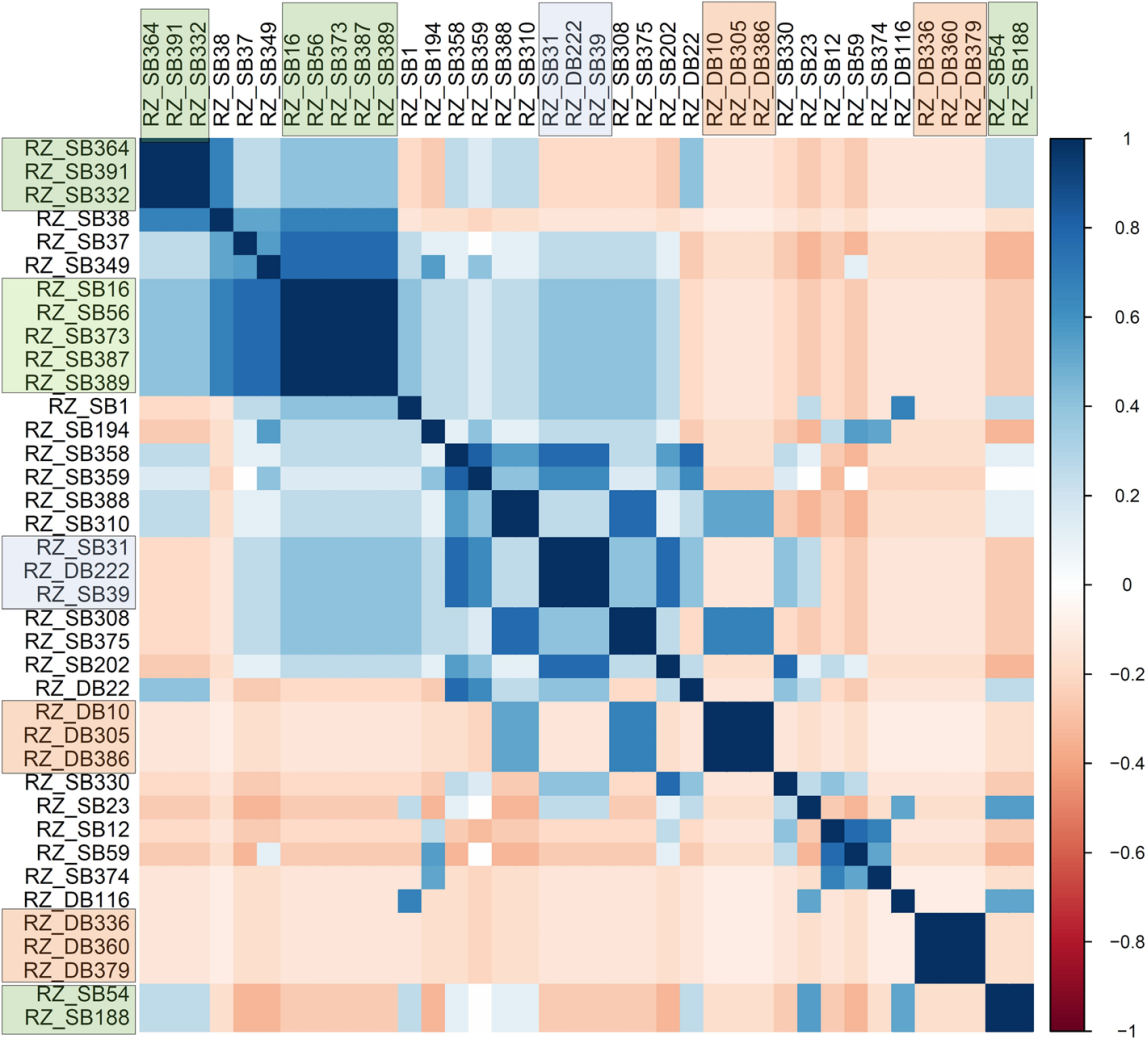
Correlation of the isolates based on their morphological characteristics. The isolates showed crop specific crop correlation. Sugar beet isolates were correlated with sugar beets and dry beans were correlated with dry beans with one exception. Correlation between sugar beets was highlighted with green, orange shades which define correlation among sugarbeet and dry bean isolates respectively. Blue shades represent inter-correlation between the sugarbeet and dry bean isolates

A total of 38 fungal colonies of the RZ_SB and RZ_DB isolates were established in laboratory and these were used for studying morphological characteristics. All the 38 isolates showed distinctive morphological variation in their appearance. Colony morphology colors varied from dark brown to light brown and light tan (Fig. 1c), sclerotia and presence of aerial hyphae (Fig. 1d, Table 1). Correlation analysis showed a positive correlation within the isolates of same crops. The isolates from sugar beet showed a positive correlation with sugar beet isolates and dry beans with dry bean isolates (Fig. 2). There was high positive correlation within the isolates of sugar beets, RZ_SB16, RZ_SB56, RZ_SB373, RZ_SB387, RZ_SB389, RZ_SB364, RZ_391, RZ_332 (*r* = 1.00 / 1.00, *p* < 0.0001). Similarly, the isolates from dry beans R_DB10, RZ-DB305, RZ_DB386, RZ_DB116, RZ_DB336, RZ_DB360, RZ_DB379 showed significantly high correlation with each other (*r* = 1.00 / 1.00, *p* < 0.0001). Though most of the isolates showed morphological correlation within the isolates of same crop there was also positive cross crop correlation with statistical significance but at a lower degree. Isolates RZ_DB22 showed correlation with RZ_SB358 (r = 0.77/1.00, *p* < 0.01) and RZ_SB359 (*r* = 0.63/1.00, *p* < 0.05), RZ_DB305 with RZ-SB375 (*r* = 0.67/1.00, *p* < 0.05) (Fig. 2) (Supplementary file: Table: T1). There was no correlation between the isolates from the same geographical origin and year of origin.

**Table 1:** Details of 41 *Rhizoctonia solani* isolates (38 test isolates and three control isolates with known AG group) with source, origin, year of isolation and morphological attributes

***Isolates***^**a**^	***Date of Isolation***	***Isolate source***	***Geographical origin***	***State***	***Morphological description***	GenBank Accession number of the ITS-rDNA
***RZ_DB22***	2001	DB	Box Butte	Nebraska	Light tan, sclerotia throughout agar	MT950064
***RZ_DB10***	2002	DB	Box Butte	Nebraska	Brown	MT950066
***RZ_DB116***	2003	DB	Scotts Bluff	Nebraska	Tan	MT950071
***RZ_DB222***	2004	DB	Scotts Bluff	Nebraska	Light tan, aerial hyphae	MT950070
***RZ_DB305***	2005	DB	Scotts Bluff	Nebraska	Brown	MT950067
***RZ_DB336***	2006	DB	Scotts Bluff	Nebraska	Light brown	MT950077
***RZ_DB360***	2007	DB	Scotts Bluff	Nebraska	Light brown	MT950078
***RZ_DB379***	2008	DB	Scotts Bluff	Nebraska	Light brown	MT950079
***RZ_DB386***	2009	DB	Scotts Bluff	Nebraska	Brown	MT950080
***RZ_SB 37***	2000	SB	Scotts Bluff	Nebraska	Dark tan, aerial hyphae, light brown sclerotia	MT950081
***RZ-SB1***	2001	SB	Scotts Bluff	Nebraska	Tan, aerial hyphae	MT950082
***RZ-SB 12***	2001	SB	Scotts Bluff	Nebraska	Smooth, cream colored	MT950074
***RZ-SB 38***	2001	SB	Scotts Bluff	Nebraska	Dark tan	MT950083
***RZ-SB 39***	2001	SB	Scotts Bluff	Nebraska	Light tan, aerial hyphae	MT950069
***RZ-SB 16***	2002	SB	Morrill	Nebraska	Dark tan, aerial hyphae	MT950085
***RZ-SB 23***	2002	SB	Scotts Bluff	Nebraska	Tan, sclerotia throughout agar, flat	MT950086
***RZ-SB 31***	2002	SB	Colorado	Colorado	Light tan, aerial hyphae	MT950063
***RZ-SB 54***	2003	SB	Scotts Bluff	Nebraska	Tan, sclerotia throughout agar, flat	MT950087
***RZ-SB 56***	2003	SB	Box Butte	Nebraska	Dark tan, aerial hyphae	MT950089
***RZ-SB 59***	2003	SB	Scotts Bluff	Nebraska	Smooth, cream-colored, brown sclerotia	MT950090
***RZ-SB 188***	2004	SB	Scotts Bluff	Nebraska	Tan, sclerotia throughout agar, flat	MT950091
***RZ-SB 194***	2004	SB	Scotts Bluff	Nebraska	Cream colored, aerial hyphae, brown sclerotia	MT950068
***RZ-SB 202***	2004	SB	Scotts Bluff	Nebraska	Smooth, light tan, aerial hyphae	MT950092
***RZ-SB 308***	2005	SB	Scotts Bluff	Nebraska	Brown, aerial hyphae	MT950093
***RZ-SB 310***	2005	SB	Scotts Bluff	Nebraska	Brown, aerial hyphae, sclerotia	MT950094
***RZ-SB 330***	2006	SB	Scotts Bluff	Nebraska	Light tan, smooth	MT950076
***RZ-SB 332***	2006	SB	Scotts Bluff	Nebraska	Dark tan, sclerotia	MT950095
***RZ-SB 349***	2006	SB	Scotts Bluff	Nebraska	Dark tan, aerial hyphae, brown sclerotia	MT950073
***RZ-SB 358***	2007	SB	Box Butte	Nebraska	Light tan, sclerotia, aerial hyphae	MT950096
***RZ-SB 359***	2007	SB	Box Butte	Nebraska	Light tan, sclerotia, aerial hyphae, brown sclerotia	MT950065
***RZ-SB 364***	2007	SB	Scotts Bluff	Nebraska	Dark tan, sclerotia	MT950063
***RZ-SB 373***	2008	SB	Scotts Bluff	Nebraska	Dark tan, aerial hyphae	MT950098
***RZ-SB 374***	2008	SB	Scotts Bluff	Nebraska	Cream colored	MT950088
***RZ-SB 375***	2008	SB	Scotts Bluff	Nebraska	Brown, aerial hyphae	MT950099
***RZ-SB 387***	2009	SB	Scotts Bluff	Nebraska	Dark tan, aerial hyphae	MT950075
***RZ-SB 388***	2009	SB	Scotts Bluff	Nebraska	Brown, aerial hyphae, sclerotia	MT950100
***RZ-SB 389***	2009	SB	Scotts Bluff	Nebraska	Dark tan, aerial hyphae	MT950101
***RZ-SB 391***	2010	SB	Wyoming	Wyoming	Dark tan, sclerotia	MT950102
***RZ_SBC23***	Unknown	SB	Imperial	Nebraska	AG group 2–2, ISG-IIIB	MT950103
***RZ_SBC28***	Unknown	SB	Swink	Colorado	AG group 4	MT950084
***RZ_SBC51***	Unknown	SB	Montana	Montana	AG group 2–2, ISG-IV	MT950072

^a^*SB* Sugar beet, *DB* Dry bean, *SBC* Sugar beet control strain

### Identity of isolates

ITS-rDNA sequence of the 38 test isolates (9 dry bean, 29 sugar beet) matched with that of 3 check isolates (*RZ_SBC23, RZ_SBC28, and RZ_SBC51*) and all these 41 sequences matched with *Rhizoctonia solani* (or *Rhizoctonia* only) based on Genebank data base (Table 1).

Infection of sugar beet seedlings by the 29 sugar beet isolates and the three check isolates (*RZ_SBC23, RZ_SBC28, and RZ_SBC51*) in the greenhouse produced similar symptom, which was similar to characteristic symptom of Rhizoctonia root rot of sugar beet (Fig. 1b). Infection of dry bean seedlings by the 9 dry bean isolates in the greenhouse produced symptom, which was similar to characteristic symptom of Rhizoctonia root rot of dry bean (Fig. 1a).

### Genetic diversity

**Fig. 3:**
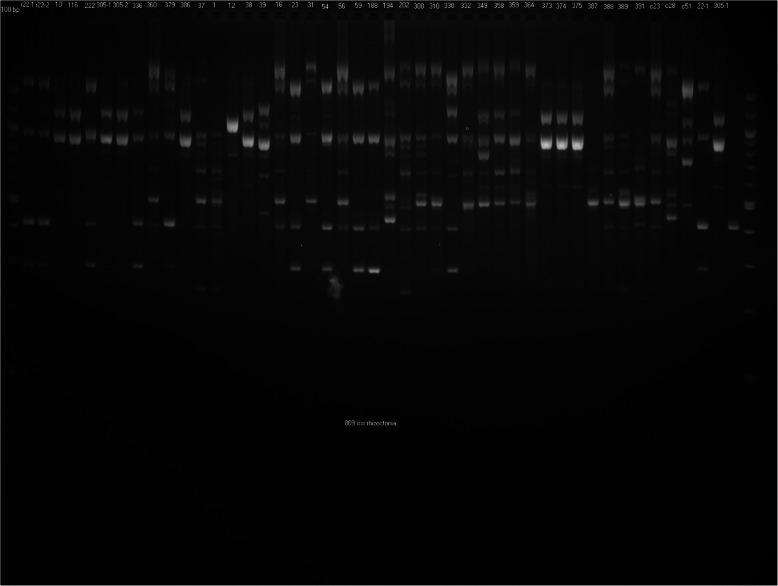
DNA marker profiles of *Rhizoctonia solani* isolates from sugar beets and dry beans with ISSR primer UBC809. Sugar beet and dry bean isolates are designated by RZ_SB and RZ_DB followed by a number

A total of 50 UBC primers were screened and 19 primers were selected based on their 100% polymorphism index. A total of 396 alleles were identified from 41 isolates (representative gel image at Fig. 3). An average number of loci per primer was 20.84 and band size ranged from 100 bp to 3 kb. The primer UBC 889 produced the highest number of polymorphic loci (29) followed by UBC 808 (27), UBC 809 (26) and UBC 812(25) (Table 2). The Shannon information index (*I)* varied from 0.235–0.280 with an average of 0.251. The percentage of polymorphic loci (% P) ranged from 43.26–92.88%. The highest % polymorphic loci (92.88%) was observed within the dry bean isolates (population 2) (Table 3). The Nei genetic distance within the population ranged from 0.033–0.083 with an average of 0.51.

**Table 2:** List of 19 ISSR markers used for the study

ISSR primer name	Sequence (5′-3′)	Marker size (bp)	Number of polymorphic markers
UBC808	AGAGAGAGAGAGAGAGC	400–2000	27
UBC809	AGAGAGAGAGAGAGAGG	200–2000	26
UBC811	GAGAGAGAGAGAGAGAC	400–3000	16
UBC812	GAGAGAGAGAGAGAGAA	300–2000	25
UBC816	CACACACACACACACAT	400–2000	15
UBC818	CACACACACACACACAG	300–2000	18
UBC821	GTGTGTGTGTGTGTGTT	400–3000	17
UBC828	TGTGTGTGTGTGTGTGA	400–3000	23
UBC835	AGAGAGAGAGAGAGAGYC	200–3000	24
UBC842	GAGAGAGAGAGAGAGAYG	100–1500	21
UBC855	ACACACACACACACACYT	300–2000	19
UBC856	ACACACACACACACACYA	400–3000	17
UBC857	ACACACACACACACACYG	300–2000	15
UBC864	ATGATGATGATGATGATG	300–2000	19
UBC880	GGAGAGGAGAGGAGA	200–2000	23
UBC884	HBHAGAGAGAGAGAGAG	200–3000	22
UBC888	BDBCACACACACACACA	200–2000	20
UBC889	DBDACACACACACACAC	200–3000	28
UBC890	VHVGTGTGTGTGTGTGT	300–2000	21

**Table 3:** Allele diversity within the population

Pop		N	Na	Ne	I	He	uHe	%P
**Pop1**	**Mean**	8.000	0.980	1.269	0.240	0.159	0.169	48.35
	**SE**	0.000	0.050	0.018	0.014	0.010	0.010	
**Pop2**	**Mean**	30.000	1.858	1.249	0.280	0.167	0.170	92.88
	**SE**	0.000	0.026	0.014	0.010	0.008	0.008	
**Pop3**	**Mean**	3.000	0.891	1.259	0.235	0.156	0.187	43.26
	**SE**	0.000	0.050	0.017	0.014	0.009	0.011	

*Na = No. of Different Alleles; Ne = No. of Effective Alleles = 1 / (p^2^ + q^2^); I = Shannon’s Information Index = −1* (p * Ln (p) + q * Ln(q)); He = Expected Heterozygosity = 2 * p * q; uHe = Unbiased Expected Heterozygosity = (2 N / (2 N-1)) * He [for Diploid Binary data and assuming Hardy-Weinberg Equilibrium, q = (1 - Band Freq.)^0.5^ and *p* = 1 – q]

**Fig. 4:**
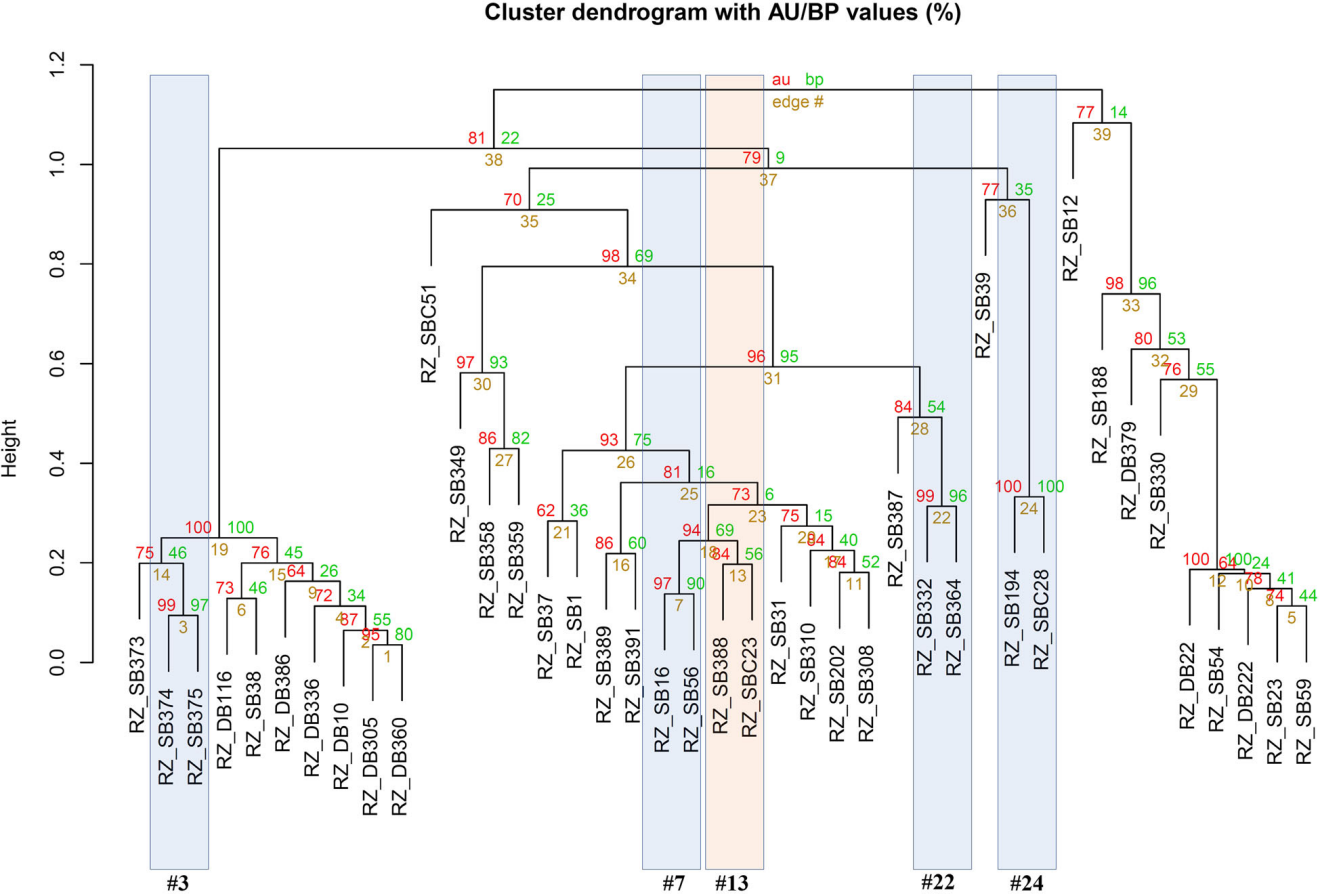
Dendrogram derived from the combined analysis of 19 ISSR primers for 41 Rhizoctonia solani with UPGMA method. In the figure the values are represented as (%); where au = approximately unbiased *p*-value (red colored), bp = bootstrap probability (bootstrap value = 1000) (green colored) and #edge = number of the sub clusters (39 total clades) (yellow colored). Clusters with Au larger than 95% are strongly supported by the data. Clades (#clade number or edge#) were grouped based on the bootstrap identity and their characteristics. Blue highlighted clades are with high bootstrap identity (≥ 80%) and statistically significant (≥ 95%). Blue highlighted (#3, #7, #22, #24) clades are with high bootstrap identity (≥ 80%) and statistically significant (≥ 95%). Organge color highlighted clade is with high bootstrap value but statistically non-significant (#13)

Cluster analysis based on the UPGMA method produced three distinct clusters. The first cluster majorly represented the isolates from dry bean (RZ_DB 116, RZ_386, RZ_DB336, RZ_DB10, RZ_DB305, and RZ_DB360) showing their genetic similarity. However, four sugar beet isolates (RZ_SB373, RZ_374, RZ_375, and RZ_SB38) also showed significant similarities with dry bean isolates in the first cluster. The second cluster included only sugar beet isolates viz. RZ_SBC51, RZ_SB349, RZ_B358, RZ_SB37, RZ-SB1, RZ_SB389, RZ_SB391, RZ_SB16, RZ_SB56, RZ_SB338, RZ_SBC23, RZ_SB359, RZ_SB387, RZ_SB332, RZ_SB364. While the third cluster showed cross relation among dry bean and sugar beet isolates (Fig. 4). Dry bean isolates like RZ_DB22, RZ_DB222, and RZ_DB379 showed genetic relatedness with sugar beet isolates RZ_SB330, RZ_SB188, and RZ_SB54 in the third cluster. Isolates like RZ_SB374 and RZ_SB375 (with au = 99% and bp = 97%) which were isolated in the year of 2008 from Scottsbluff showed similarity in genetic makeup. Isolates like RZ_SB332 (2005) showed similar genetics with isolates RZ_SB364 (2006) (au = 99% and bp = 96%) (#22).

The AMOVA analysis based on ΦPT values indicated that most of the genetic diversity occurred within the population (87%, *P* < 0.023) while variability among the population only contributed 13% (*P* < 0.023) (Table 4). Statistically significant genetic differentiation was observed among the isolates.

**Table 4:** Hierarchical distribution of genetic diversity among the population of *R. solani* from sugar beet and dry beans

Source	df	SS	MS	Estimated Variation	% of variation	ΦPT^a^	p
**Among Pops**	2	219.93	109.97	7.29	13%	0.14	< 0.023
**Within Pops**	38	1787.53	47.04	47.04	87%		
**Total**	40	2007.46		54.33	100%		

^a^ΦPT = AP / (WP + AP) = AP / TOT (Key: AP = Est. Var. Among Pops, WP = Est. Var. Within Pops)

## Discussion

The genus *Rhizoctonia* is a diverse group of fungus which causes stem and root rot as well as foliar blights in many crops [1]. *Rhizoctonia solani* Kuhn, a ubiquitous soil borne basidiomycete, which causes diseases in many economically important crops like rice, potato, soybean, corn, sugar beet and dry beans [22]. In the classical identification method, *R. solani* was characterized based on differences in pathogenicity, morphology, and physiology [18]. In this study, a total of 38 *R. solani* was isolated from sugar beet and dry bean in a time span of 10 years from different location of western Nebraska, USA (Table 1). Studies on cultural characteristics revealed that the colony color of the different *R. solani* isolates varied from cream-colored to brown, dark tan to light tan in PDA culture plates with the production of areal hyphae and sclerotia with dark to light brown color (Table 1). The results showed a close agreement with other works [11, 23, 24].

**Fig. 5:**
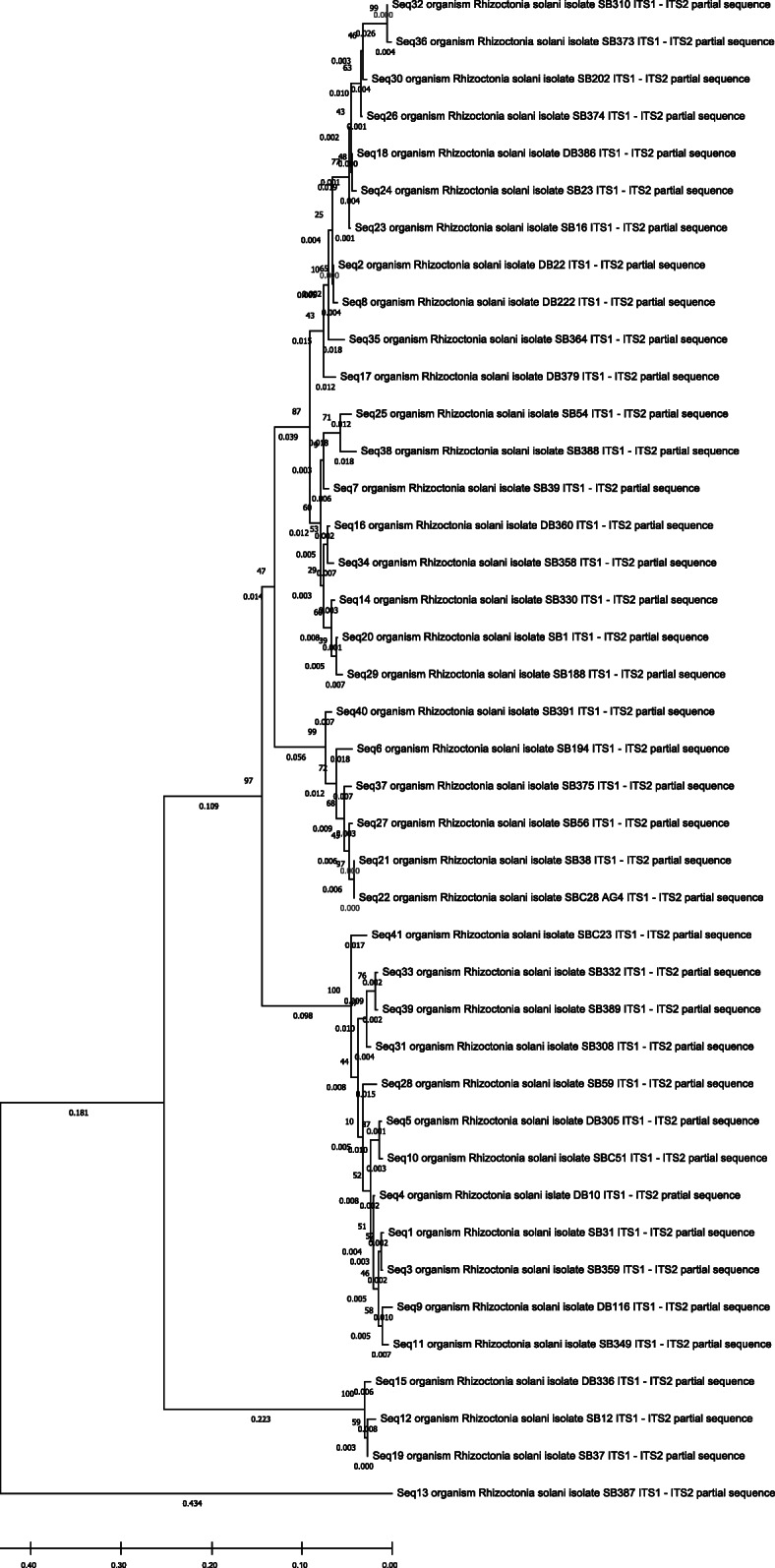
The evolutionary history of the 41 isolates based on ITS-rDNA sequences was inferred using the Neighbor-Joining method. The optimal tree with the sum of branch length = 1.62 is shown. The percentage of replicate trees in which the associated taxa clustered together in the bootstrap test (1000 replicates) are shown next to the branches (next to the branches). The evolutionary distances were computed using the Jukes-Cantor method and are in the units of the number of base substitutions per site

Results of the genetic analysis indicated a high degree of genetic diversity within the population (87%). Both the population of sugar beet and dry bean have unique genetic makeup which can be observed from the marker genotypes. The sugar beet and dry bean isolates are mostly conserved within each crop and formed distinct clusters in dendrogram analysis with a few variations. Some of the sugar beet isolates also showed cross correlation with the isolates of dry bean (cluster 1 and cluster 3) (Fig. 4). This suggests relatedness among the population and wide pathogenicity spectrum of the group. AMOVA analysis also showed low variation among population (13%) compared to within the population (87%). PCA analysis and grouping based on marker genotypes also showed similar grouping patterns (Supplementary File: Fig. S1). It indicates, however, the origin of *R. solani* for sugar beet and dry bean are same but there is a certain degree of differentiation. The difference may have originated during the evolution and selection over pathogenicity. Similar results were observed form the studies of Dubey et al., (2012), where the *R. solani* isolates were independent or did not correspond to crops of origin [10, 25]. Sequence based phylogenetic study also showed wide diversity and independent nature of the pathogens. The isolated pathogens grouped into clusters without corresponding to their host crop (Fig. 5).

Morphological classification of the isolates showed high level of differentiation among the isolates from sugar beet and dry bean. Grouping based on only morphological traits showed correlation within the isolates of same population (Fig. 2). However, marker-based analysis showed certain degree of cross-correlations among the populations. Grouping of the isolates based on location and year highlighted stimulating facts. High bootstrap (97%) and *p*-value (99%) between the isolates RZ_SB374 and RZ-375 indicate the same genetic origin based on the fact they were collected in same year (2008) and from same place (Scottsbluff) (Fig. 4). It also indicates the chances of them being in the same pathovar group which couldn’t be morphologically differentiated. Isolates from consecutive years also showed a high degree of genetic similarity defining the chances of same pathogens infecting the fields in the successive year. This also indicates their inoculum may have been present in the crop residues or soil from previous year, which are left untreated and produced the infection. Isolates like RZ_SB194 showed 100% bootstrap identity with control strain RZ_SBC28 of sugar beet (au =100%). This suggests genetic similarity and possibility of RZ_194 belonging to AG-Group 2–2 IIIB, which is a major anastomosis group responsible for sugar beet crown and root rot [26]. Isolates RZ_SB388 and control strain RZ_SBC23 also showed genetic resemblance but with low bootstrap probability value (56%) and was not statistically significant (84%) (#13) (Fig. 4). It can be concluded, though there is some degree of similarity, they are totally different pathovars.

**Fig. 6:**
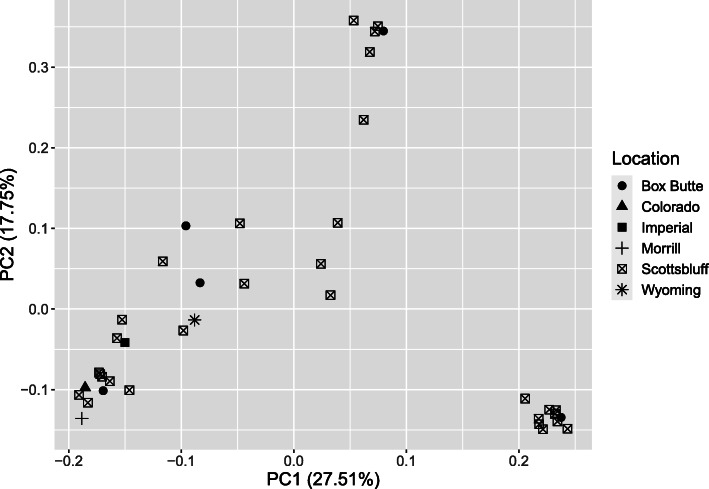
Location-based distribution of the *R. solani* isolates based on marker data

The location-based grouping of the isolates based on marker profile showed random distribution. Scottsbluff with the highest number of isolates showed a correlation with all the isolates from other places and among themselves. Isolates from the Scottsbluff showed a high degree of similarity with the isolates of Box Butte, Morril, Imperial. Although sampling of this study was uneven with respect to crop and collection. But, there was a mixed genetic population as noticed in cluster analysis. Isolates distributed independently of their geographical locations. Among the 31 isolates from Scottsbluff, there were four distinct divisions (Fig. 6). Several studies reported similar uneven relationship or no correlation between the place of origin and isolates of *R. solani* [10, 25, 27, 28].

Relying only on morphological characteristics often may results in misidentification as the pathogens belonging to same group with similar morphological feature may differ in pathogenicity. In this study, we observed morphological correlation showed a distinct difference between the isolates of sugar beet and dry bean, while genetic diversity showed certain degree of cross correlation between the ioslates of sugar beet and dry bean. Therefore, using morphological traits as sole identification method can result in biased grouping when the population are distinct or unique in their genetic makeup. Thus, this study proved that marker information in addition to morphological traits gave a better identification and characterization of intra and intergenic genetic variations. For differentiation and characterization of the *R. solani* from sugar beet and dry bean, ISSR marker was found suitable with the morphological features. This method can give a comprehensive estimation of genetic diversity of polyphyletic isolates where anastomosis or morphological features were not sufficient. Identification and proper categorization of the pathogen will be helpful in designing integrated disease management guidelines for sugar beet and dry beans of mid western America.

## Conclusion

There were no significant genetic and morphological differences in both sugar beet and dry bean isolates which were collected over 10 years in Nebraska Panhandle. This suggested that possibly there was no significant genetic mutations occurred during these years, which would otherwise cause challenges to existing disease management strategies. Majority of the isolates from sugar beet showed nearby association within the population. However, ISSR-DNA marker-based similarity among a few isolates of both the crops suggesting their broad pathogenicity. This may need to be confirmed by cross-pathogenicity tests where the same isolate is used to infect both sugar beet and dry bean. Identification and characterization of the pathogen may be helpful in designing integrated disease management guidelines for sugar beet and dry bean of mid western Nebraska.

## Methods

### Fungal isolates

Infected plants were collected from different regions of western Nebraska (Fig. 1a - b). A total of 38 *R. solani* were isolated from the collected samples and used for this study. Twenty-nine were from sugar beet and nine isolates were from dry bean. Isolates were recovered from symptomatic diseased sugar beet and dry bean roots. For isolation, surface sterilized plant material was platted on potato dextrose agar (PDA) medium containing streptomycin antibiotic to reduce the opportunistic bacterial growth. Culture plates were incubated at 26°C. Isolates were identified microscopically, and morphological features were recorded (Table. 1) [29]. Three control strains of *R. solani* of sugar beet (RZ_SBC23, RZ_SBC28, and RZ-SBC51) from Nebraska, Montana and Colorado were collected from Dr. Linda E. Hanson (USDA – ARS Sugarbeet and Bean Research, East Lansing, Michigan) (Table 1). These strains were used for ISSR marker analysis to compare the genetic relatedness among the isolates and with known AG grouping. Population 1, 2, and 3, were used in this report to indicate the group of 29 sugar beet isolates, 9 dry bean isolates, and 3 control strains.

### Validation of isolates identify

Isolated pathogens were taxonomically validated by sequencing ITS1 (TCCGTAGGTGAACCTGCGG) and ITS2 (GCTGCGTTCTTCATCGATGC) region. Identity was confirmed by sequence similarity search with NCBI Blast with nr/nt nucleotide database. The sequences were submitted to GenBank for accession number (MT950063-MT950103) and further references (Table 1).

Pathogenicity test was done in greenhouse following established methods being used in Harveson lab using the isolated fungal culture of the nine RZ_DB isolates on the host plant dry bean and 29 RZ_SB on the host plant sugar beet.

### DNA extraction

For DNA extraction fresh fungal culture was used. Five ¼ x ¼ inch plugs of agar were placed into 50 ml of sterile PDB and grown for 5 days at 26 °C. After the incubation period, mycelia were harvested by filtration through cheesecloth. The collected mycelia were lyophilized with liquid nitrogen and grounded into a fine powder with mortar and pestle. The powder was transferred to 50 ml conical tubes containing 15 ml of CTAB extraction buffer (2% CTAB, 1.4 M NaCl, 20 mM EDTA, pH 8.0, 0.1 M Tris, pH 8.0, 0.4% B-mercaptoethanol) preheated to 65 °C. The samples were then incubated in a 65 °C water bath for 1 h, with mixing at an interval of every 10 min. Samples were cooled for 10 min and 20 ml of chloroform/isoamyl alcohol (24:1) was added and mixed with each tube. The tubes were centrifuged at 3500 rpm for 20 min at 15 °C. The aqueous layer was transferred to a new tube and a double volume of chilled 95% ethanol was added to precipitate the DNA. The tubes were centrifuged at 3500 rpm for 1 min and the supernatant was discarded. Pellets were washed with chilled 70% ethanol. After all, ethanol was removed the dry DNA pellet was suspended in 1 ml of TE buffer (10 mM Tris-HCl and 1 mM EDTA, pH 8.0) and treated with 5 ul of RNAse (10 mg/ml) at room temperature for 1 h. DNA was quantified using the mini-gel method [30] that compares band intensities with a standard lambda/HindIII DNA marker (Gibco BRL, Betheseda, MD) in a 0.8% agarose gel. DNA was diluted to a concentration of 25–50 ng/ul for use in a polymerase chain reaction (PCR).

### PCR amplification of ISSR

The 50 UBC primers screened in this study were obtained from Eurofins Genomics (Huntsville, Alabama). Nineteen (Table 2) were selected for analysis based on amplification profile (band intensity, quality, and reproducibility in at least two independent replications) on two random isolates. PCR amplification was performed in a 25 μl reaction mixture containing Promega 5x Green GoTaq Flexi Buffer, 2 mM MgCl_2_, 100 μm of dNTPs, 0.24 μM primer, 50–100 ng DNA, and 1 unit of *Taq* polymerase. The cyclic reaction was set at initial denaturation at 94 °C for 30 s, annealing at 50 °C for 45 s, and an elongation at 72 °C for 2 min (45 cycles). Final elongation was performed for 10 min. The PCR was completed on an Applied Biosystems Thermocycler 2720. PCR products were separated electrophoretically in 2% agarose gels including 0.5 μg/ml ethidium bromide and bands were visualized in the gel-doc system (Biorad, USA). ISSR gels were photographed using FOTO Analyst Express Electronic Imaging System (Fotodyne, Inc). Marker size range was determined by comparison with a 100 bp DNA ladder (New England BioLabs). When scoring the gels, a marker locus was considered polymorphic if the band was not present in every isolate. Only clear DNA bands that were reproducible were scored. ISSR marker loci were designated by primer name.

### Data analysis

A correlation matrix and dendrogram were prepared with unweighted pair group method with arithmetic mean (UPGMA) method to distinguish the isolates based on morphological characteristics. The categorical data of morphological traits were converted into a 0, 1 matrix based on presence and absence of that. The dendrogram (method = UPGMA) and correlation plot (method = Pearson correlation) were prepared using R statistical software (package: pvclust and corrplot). Dominant polymorphic ISSR markers were scored based on presence and absence with 0 (absent) - 1 (present) matrix. Cluster analysis was carried out using UPGMA method and dendrogram was created by using R statistical software with a bootstrap value of 1000 with package pvclust [31]. The package was used to compute two values: an approximately unbiased (AU) *p-*value based on multi-step multiscale bootstrap resampling procedure [32] and a bootstrap probability (BP) *p-*value from ordinary bootstrap resampling [33]. A significance threshold of *α* = 0.05 (95% confidence interval) was used in this approach. For a cluster with AU *p*-value > 0.95 (95%), the hypothesis that “the cluster does not exist” is rejected with significance level 0.05. The percentage of polymorphic loci (P), Shannon’s diversity index (*I*) and Nei’s gene diversity were calculated to estimate the genetic variation among the isolates using GenAlex 6.5 software [34]. Genetic differentiation among populations was estimated by pairwise values of *ф*_PT_. Analysis of molecular variance (AMOVA) was used to compute the genetic variation among and within the population. AMOVA calculations were performed in GenAlex 6.5. Principle component analysis was used to determine genetic differentiation among the isolates of different region using R statistical software (Package: FactoMiner, factoextra, and ggplot2). Morphological and genetic traits were correlated with R-stat to determine the diversity among and within the isolates of sugar beets and dry beans (Package: corrplot). For phylogenetic analysis, a neighbor-joining tree was constructed using bootstrapping method (1000) and evolutionary distance was computed using Jukes-Cantor method MEGA-X software.

## Supplementary Information

**Additional file 1: Figure S1.** Principal component analysis based on (a) Morphological traits, (b) Marker profile. Morphological traits doesn’t able to completely distinguish between the two population and they formed one big cluster while marker profile showed to distinct group for two populations.

**Additional file 2: Table S1.** Correlation Matrix_Morphology.

## Data Availability

All data generated or analyzed during this study are included in this published article [and its supplementary information files].

## Abbreviations

AMOVA: Analysis of molecular variance
RZ_DB: Rhizoctonia_Dry Bean
RZ_SB: Rhizoctonia_Sugar Beet
RZ_SBC: Rhizoctonia_Sugar Beet Control
bp: Base pair
