# Supplementary figures and images for: A longitudinal study on morpho-genetic diversity of pathogenic Rhizoctonia solani from sugar beet and dry beans of western Nebraska
Source: BMC Microbiol. 2020 Nov 17;20:354. doi: 10.1186/s12866-020-02026-9 (PMC7672822; doi:10.1186/s12866-020-02026-9)

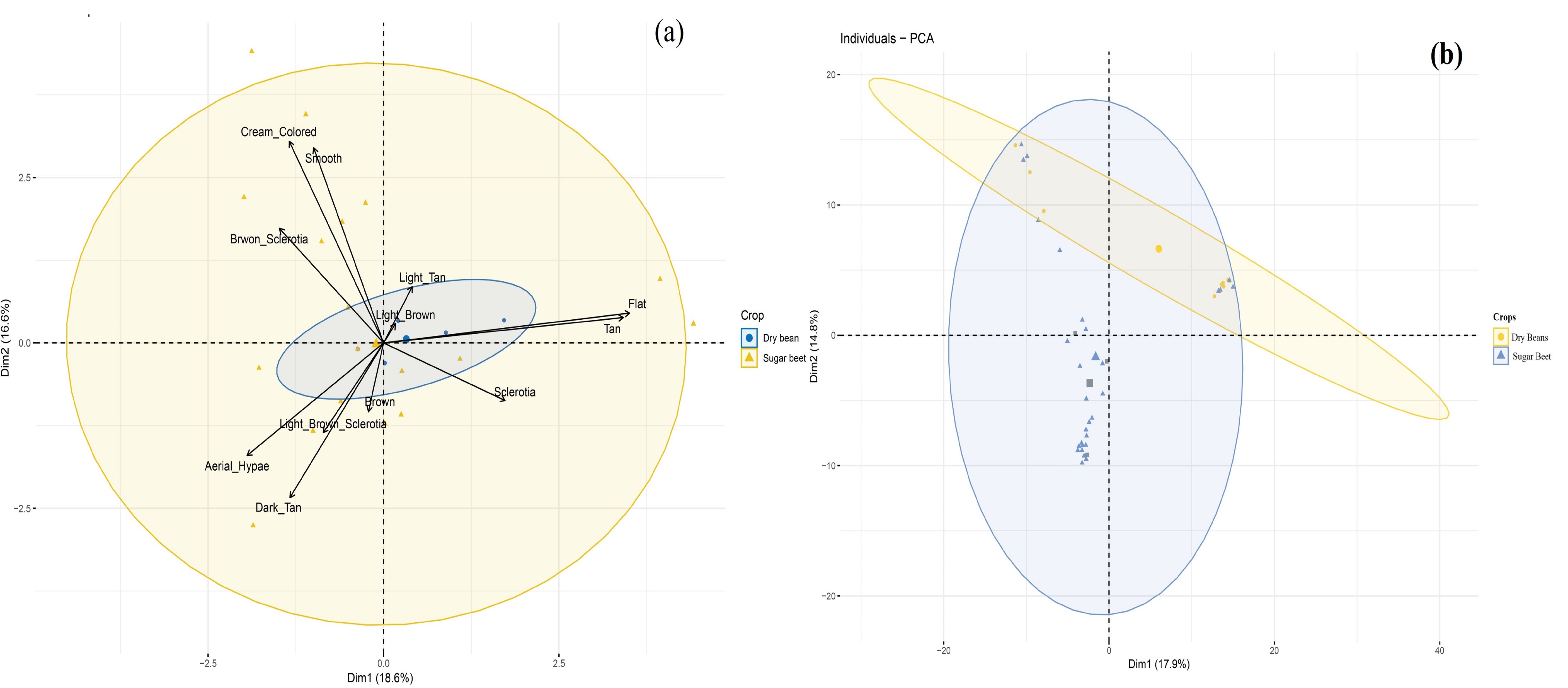

Supplement: Supplementary file 1 — Additional file 1: Figure S1. Principal component analysis based on (a) Morphological traits, (b) Marker profile. Morphological traits doesn’t able to completely distinguish between the two population and they formed one big cluster while marker profile showed to distinct group for two populations. [file 12866_2020_2026_MOESM1_ESM.jpg]
